# Nitrosative damage to free and zinc-bound cysteine thiols underlies nitric oxide toxicity in wild-type *Borrelia burgdorferi*

**DOI:** 10.1111/j.1365-2958.2011.07691.x

**Published:** 2011-05-19

**Authors:** Travis J Bourret, Julie A Boylan, Kevin A Lawrence, Frank C Gherardini

**Affiliations:** Laboratory of Zoonotic Pathogens, Rocky Mountain Laboratories, National Institute of Allergy and Infectious Diseases, National Institutes of Health903 South 4th Street, Hamilton, MT 59840, USA.

## Abstract

*Borrelia burgdorferi* encounters potentially harmful reactive nitrogen species (RNS) throughout its infective cycle. In this study, diethylamine NONOate (DEA/NO) was used to characterize the lethal effects of RNS on *B. burgdorferi*. RNS produce a variety of DNA lesions in a broad spectrum of microbial pathogens; however, levels of the DNA deamination product, deoxyinosine, and the numbers of apurinic/apyrimidinic (AP) sites were identical in DNA isolated from untreated and DEA/NO-treated *B. burgdorferi* cells. Strains with mutations in the nucleotide excision repair (NER) pathway genes *uvrC* or *uvrB* treated with DEA/NO had significantly higher spontaneous mutation frequencies, increased numbers of AP sites in DNA and reduced survival compared with wild-type controls. Polyunsaturated fatty acids in *B. burgdorferi* cell membranes, which are susceptible to peroxidation by reactive oxygen species (ROS), were not sensitive to RNS-mediated lipid peroxidation. However, treatment of *B. burgdorferi* cells with DEA/NO resulted in nitrosative damage to several proteins, including the zinc-dependent glycolytic enzyme fructose-1,6-bisphosphate aldolase (BB0445), the B*orrelia* oxidative stress regulator (BosR) and neutrophil-activating protein (NapA). Collectively, these data suggested that nitrosative damage to proteins harbouring free or zinc-bound cysteine thiols, rather than DNA or membrane lipids underlies RNS toxicity in wild-type *B. burgdorferi*.

## Introduction

*Borrelia burgdorferi,* the agent of Lyme disease, has a complex life cycle that involves survival in various mammalian hosts and transmission between these hosts by its arthropod vector (*Ixodes scapularis*). Reactive oxygen species (ROS) [e.g. superoxide (O_2_•−), hydrogen peroxide (H_2_O_2_) and hydroxyl (OH•)] and reactive nitrogen species (RNS) [e.g. nitric oxide (NO), nitrogen dioxide (NO_2_•), nitrous anhydride (N_2_O_3_) and peroxynitrite (ONOO−)] produced in tick organelles (e.g. salivary glands), or by immune cells in mammalian hosts could present significant obstacles to *B. burgdorferi* during this cycle. Surprisingly, *B. burgdorferi* harbours a very small repertoire of antioxidant defences. These include a low-molecular-weight thiol, coenzyme A, as well as antioxidant/redox proteins coenzyme A disulphide reductase (BB0728), neutrophil activating protein (BB00690), sulphoxide reductase (BB0340), superoxide dismutase (BB0153), thioredoxin (BB0061) and thioredoxin reductase (BB0515) (Fraser *et al*., 1997; Boylan *et al*., 2006; Esteve-Gassent *et al*., 2009). The lack of intracellular iron (< 10 atoms per cell) and proteins that utilize iron as a cofactor (Fe–S clusters, cytochromes, etc.) likely contributes to the innate resistance of *B. burgdorferi* cells to some oxidative damage since the potential for Fenton chemistry appears to be very limited (Fraser *et al*., 1997; Posey and Gherardini, 2000). This has been demonstrated experimentally since *B. burgdorferi* DNA has been shown to be highly resistant to oxidative damage following ROS treatment *in vivo* (Boylan *et al*., 2008). Host-derived polyunsaturated fatty acids, enriched in membrane lipids and lipoproteins, are highly susceptible to peroxidation and appear to be the primary targets of ROS in *B. burgdorferi* (Boylan *et al*., 2008). Nevertheless, through the combination of its small arsenal of antioxidant defences and the lack of intracellular targets, *B. burgdorferi* appears to be sufficiently protected against the lethal damage typically inflicted by ROS.

While the effects of ROS on *B. burgdorferi* cells are becoming clear, little is known about how RNS target *B. burgdorferi* cells. RNS are involved in host defences against a broad range of microbial pathogens. RNS-dependent cytotoxicity encompasses oxidative, nitrative and nitrosative modification or damage to an array of biomolecules including DNA, protein thiols, secondary amines, tyrosine residues, cytochromes, thiol-bound metals and polyunsaturated fatty acids of lipid membranes (Fang, 2004). The mammalian inducible nitric oxide synthase (iNOS) is the primary source of NO involved in innate host defences, although circulating and enterosalivary nitrite and nitrate also serve as reservoirs of bioavailable RNS (Lundberg *et al*., 2008). Blood-sucking arthropods exploit the vasodilating activity of NO and harbour diverse mechanisms to deliver RNS to their host. For example, salivary glands of the dog tick *Dermacentor variabilis* harbour NOS activity (Bhattacharya *et al*., 2000), while the kissing bug *Rhodnius prolixus* delivers NO to its host through haem molecules referred to as nitrophorins (Ribeiro *et al*., 1993; Ribeiro and Walker, 1994). The genome of the primary vector of Lyme disease *I. scapularis* (GenBank Accession No: NZABJB000000000) encodes a putative NOS that may facilitate the production of NO in the salivary glands; however, the presence of nitrophorins have yet to be described in this tick species. It seems likely that *B. burgdorferi* cells encounter RNS during most stages of the infective cycle.

Previous studies have demonstrated that *B. burgdorferi* is susceptible to cytostasis or killing by high concentrations (2.5–10 mM) of NO donor compounds *S-*nitroso-*N*-acetylpenicillamine or *S*-nitrosoglutathione and by iNOS-derived NO from macrophages (Ma *et al*., 1994; Modolell *et al*., 1994; Lusitani *et al*., 2002). However, the mechanism(s) by which NO or its oxidative congeners (i.e. RNS) exert antimicrobial activity on this pathogen have not been investigated. The following study was conducted to identify the biological targets of RNS in *B. burgdorferi*. Unlike ROS (Boylan *et al*., 2008), RNS do not catalyse peroxidation of polyunsaturated fatty acids of *B. burgdorferi* cell membranes nor compromise the integrity of the cell envelope. Additionally, RNS-dependent DNA damage was absent in cells exposed to lethal concentrations of diethylamine NONOate (DEA/NO) due in part to the activity of the nucleotide excision repair (NER) pathway. Rather, both free and zinc-bound cysteine thiols of proteins including the glycolytic enzyme fructose-1,6-bisphosphate aldolase (BB0445), the transcriptional regulator BosR and the membrane protein NapA were identified as the primary targets of RNS in *B. burgdorferi*.

## Results

### *B. burgdorferi* is susceptible to killing by RNS produced by DEA/NO

**Fig. 1:**
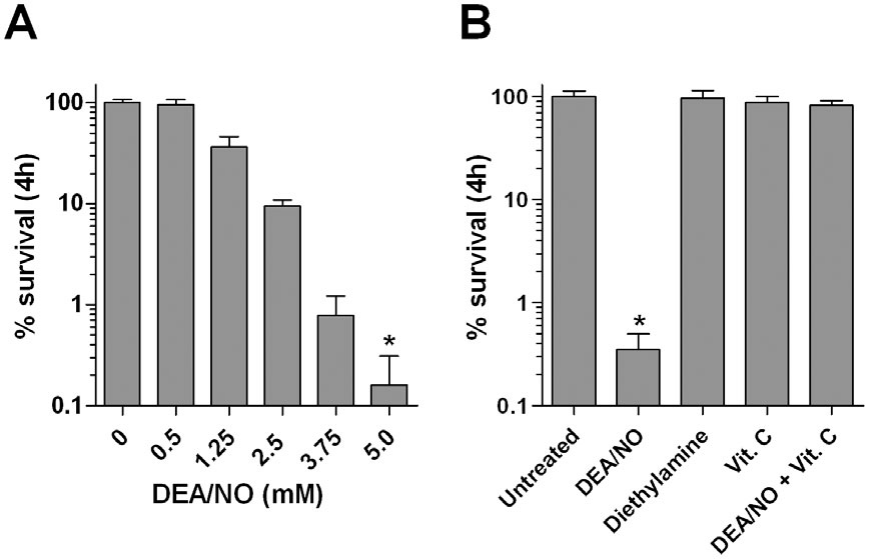
*B. burgdorferi* is susceptible to killing by RNS. A. *B. burgdorferi* cells were challenged with various concentrations of DEA/NO ranging from 0 to 5.0 mM under microaerobic conditions for 4 h. The % of surviving cells was determined by enumerating cfu following growth on P-BSK. The data represent the mean ± SD of three independent experiments. *P < 0.01 compared with untreated controls. B. Per cent survival was determined for *B. burgdorferi* cells exposed to 5.0 mM DEA/NO, 5.0 mM diethylamine and/or 10 mM ascorbic acid (Vit. C) for 4 h. The data represent the mean ± SD of three independent experiments. *P < 0.01 compared with untreated controls.

*Borrelia burgdorferi* is susceptible to cytostasis or killing by RNS generated from NO donors and iNOS-derived NO produced by bone marrow-derived macrophages (Ma *et al*., 1994; Modolell *et al*., 1994; Lusitani *et al*., 2002). In contrast, *B. burgdorferi* is highly resistant to killing by ROS. While polyunsaturated fatty acids of *B. burgdorferi* lipid membranes have been identified as the primary targets of ROS, the biological targets of RNS remain to be identified. In order to determine the biological targets of RNS, the bactericidal activity of the NO donor DEA/NO towards *B. burgdorferi* was examined using late-log-phase spirochaetes challenged under microaerobic (3% O_2_, 5% CO_2_) conditions. The antimicrobial activity of DEA/NO against *B. burgdorferi* was dose-dependent, with ∼ 99.9% of cells killed by 5.0 mM DEA/NO (Fig. 1A). Killing by DEA/NO was due to the release of NO because treatment of cells with diethylamine alone was not bactericidal (Fig. 1B). Moreover, the addition of the antioxidant ascorbic acid (vitamin C), an efficient scavenger of RNS (Licht *et al*., 1988) completely protected microaerobic cultures of *B. burgdorferi* from killing by 5.0 mM DEA/NO (Fig. 1B). Collectively, these data show the antimicrobial activity of DEA/NO against *B. burgdorferi* and establish the conditions necessary to study the mechanisms underlying RNS-dependent toxicity.

### RNS do not promote oxidation of *B. burgdorferi* membrane lipids

**Fig. 2:**
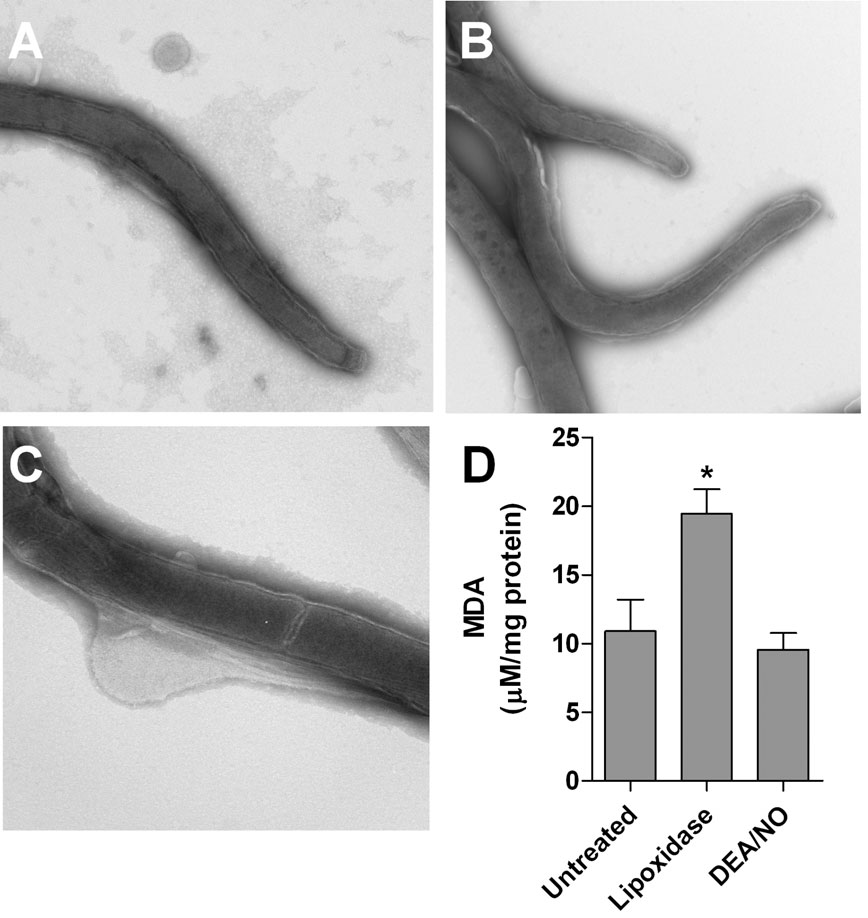
RNS had no effect on *B. burgdorferi* cell membranes. A–C. Electron micrographs are shown for untreated (A), 2.5 mM DEA/NO-treated (B) and lipoxidase-treated (C) *B. burgdorferi* cells. D. The quantities of malondialdehyde (MDA) were determined for *B. burgdorferi* cells cultured in the presence or absence of lipoxidase (1 mg ml^−1^) or 5.0 mM DEA/NO for 4 h. MDA concentration is represented as the mean ± SD of three biological samples. *P < 0.002 compared with untreated controls.

Previously, polyunsaturated fatty acids of membrane lipids and lipoproteins were identified as the primary targets of ROS in *B. burgdorferi* (Boylan *et al*., 2008). The fact that NO accumulates and undergoes an accelerated rate of autooxidation in the hydrophobic space of lipid membranes (Moller *et al*., 2005), raised the possibility that the antimicrobial activity of DEA/NO may be due to membrane damage. However, examination of untreated and 2.5 mM DEA/NO-treated *B. burgdorferi* cells by electron microscopy revealed that both groups of cells harboured intact cell membranes (Fig. 2A and B), while only lipoxidase-treated cells displayed membrane damage indicative of lipid peroxidation (Fig. 2C and Boylan *et al*., 2008). Measurements of malondialdehyde (MDA), a stable breakdown product of polyunsaturated fatty acid peroxides and a surrogate marker for lipid peroxidation, supported the observations made by electron microscopy since *B. burgdorferi* cells exposed to 5.0 mM DEA/NO yielded similar concentrations of MDA (9.6 µM mg^−1^) as untreated controls (10.9 µM mg^−1^ protein) (Fig. 2D). In accordance with the membrane damage elicited by lipoxidase (Fig. 2C), identically treated cells yielded a twofold increase in MDA (19.5 µM per mg protein) compared with untreated cells (Fig. 2D). Therefore, the antimicrobial activity of DEA/NO on *B. burgdorferi* was independent of membrane damage caused by lipid peroxidation. However, these data do not preclude the formation of RNS-catalysed nitrosative or nitrative lipid modifications in DEA/NO-treated *B. burgdorferi* cells.

### *B. burgdorferi* DNA is resistant to RNS-dependent genotoxicity

**Fig. 3:**
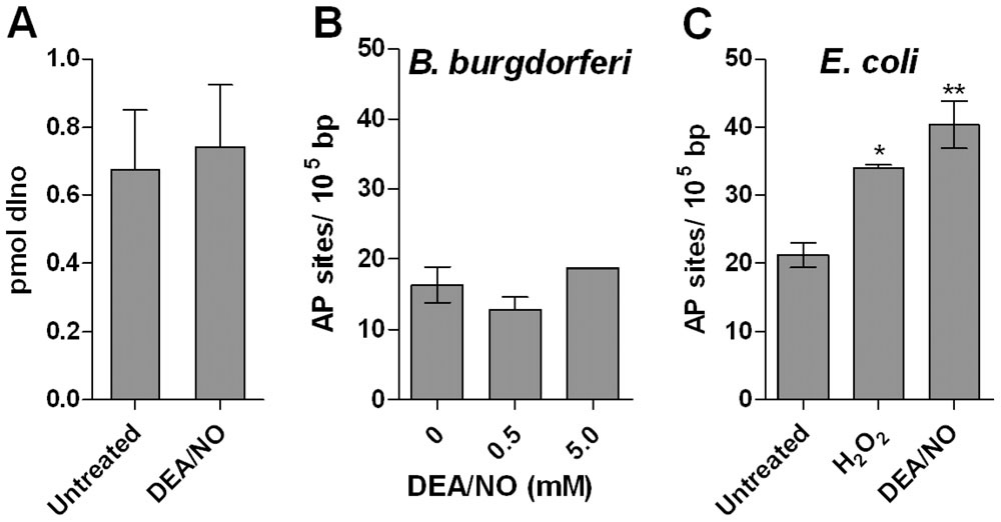
Effects of RNS on *B. burgdorferi* DNA. A. The amount of deoxyinosine (dIno) was measured in DNA harvested from untreated and *B. burgdorferi* cultures treated with 5.0 mM DEA/NO for 4 h. B. The number of apurinic/apyrimidinic (AP) sites were quantified in DNA harvested from *B. burgdorferi* cultures exposed to 0, 0.5 and 5.0 mM DEA/NO for 4 h. Data represent the mean ± SD of three biological samples. C. The number of AP sites were measured in DNA harvested from Δ*ahpCF E. coli* challenged with 2.5 mM H_2_O_2_ or 5.0 mM DEA/NO for 4 h. Data represent the mean ± SD of three biological samples. *P < 0.001 compared with untreated controls; **P < 0.005 compared to untreated controls.

ROS- and RNS-dependent antimicrobial activity is often attributed to direct or indirect DNA damage. *B. burgdorferi* DNA is resistant to oxidative damage *in vivo* (Boylan *et al*., 2008), which is likely the consequence of the lack of intracellular iron and iron-dependent proteins used to drive the formation of OH^•^ by the Fenton reaction. In contrast to some ROS, RNS are capable of directly damaging DNA independent of Fenton chemistry. For example, N_2_O_3_ catalyses hydrolytic deamination of DNA bases leading to depurination and subsequent DNA strand breakage *in vitro* (Wink *et al*., 1991). To determine whether RNS catalysed deamination of *B. burgdorferi* DNA, the concentrations of deoxyinosine, the product of deoxyadenosine deamination, were compared in *B. burgdorferi* left untreated versus cells exposed to 5.0 mM DEA/NO under microaerobic conditions. DNA harvested from untreated *B. burgdorferi* yielded identical levels of deoxyinosine as spirochaetes treated with 5.0 mM DEA/NO (Fig. 3A), suggesting that the lethal effects of RNS were independent of DNA deamination.

The base excision repair pathway (BER) repairs several types of DNA lesions including deaminated bases. Apurinic/apyrimidinic (AP) sites generated by an intermediate step in the BER pathway are reliable signatures of both oxidative and nitrosative DNA damage. Therefore, the ability of DEA/NO to stimulate AP site formation in *B. burgdorferi* was tested. *B. burgdorferi* cells treated with 0.5 mM or 5.0 mM DEA/NO yielded similar numbers of AP sites in their DNA as untreated controls (Fig. 3B, left panel), which corresponded to the lack of RNS-catalysed DNA deamination seen in Fig. 3A. Conversely, treatment of a ROS/RNS-sensitive *Escherichia coli* strain TA4315 (Δ*ahpCF*) with 2.5 mM H_2_O_2_ or 5.0 mM DEA/NO resulted in increased numbers of AP sites in DNA compared with untreated controls (Fig. 3B, right panel). Collectively, these data show that, unlike *ahpCF*-deficient *E. coli*, RNS toxicity in *B. burgdorferi* occurs without a detectable increase in DNA damage.

### The NER pathway repairs *B. burgdorferi* DNA damaged by RNS

**Fig. 4:**
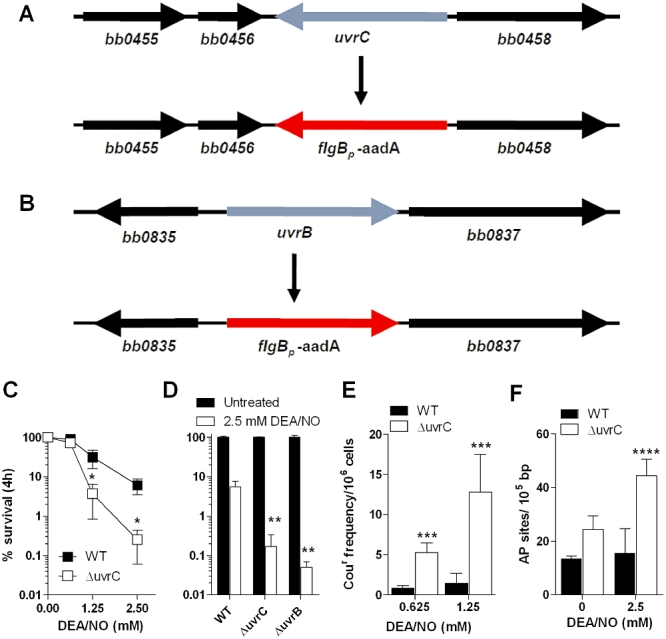
RNS-dependent damage to *B. burgdorferi* DNA is repaired by the nucleotide excision repair pathway. A. A diagram is shown for the deletion of *uvrC* (*bb0457*) with a *flgB_p_::aadA* resistance cassette. The NER gene *uvrC* (blue) was replaced by allelic exchange using a suicide vector harbouring a streptomycin resistance cassette under the control of a *flgB* promoter (red) flanked by the 1 kb upstream and downstream regions (black) of *uvrC* as described in *Experimental procedures*. B. A diagram is shown of the deletion strategy for *uvrB* (*bb0836*) and replacement with a *flgB_p_::aadA* resistance cassette using the strategy outlined in (A). C. B31 A3 (WT) and B31 A3 Δ*uvrc::aadA* (Δ*uvrC*) cells were treated with 0 mM, 0.625 mM, 1.25 mM and 2.5 mM DEA/NO under microaerobic conditions for 4 h. The % survival was determined by enumerating cfu following plating on P-BSK. *P < 0.005 compared with untreated controls. D. B31 A3 (WT), B31 A3 Δ*uvrC::aadA* (Δ*uvrC*) and B31 A3 Δ*uvrB::aadA* (Δ*uvrB*) cells were treated with 0 mM or 2.5 mM DEA/NO under microaerobic conditions for 4 h. Per cent survival was determined by enumerating cfu following plating on P-BSK. **P < 0.005 compared with wild-type controls. E. The spontaneous resistance frequency to 250 ng ml^−1^ coumermycin A was determined for B31 A3 (WT) and B31 A3 Δ*uvrc::aadA* (Δ*uvrC*) cells treated with 0.625 mM or 1.25 mM DEA/NO for 4 h. The data represent the mean ± SD of three independent experiments. ***P < 0.005 compared with untreated controls. F. The number of AP sites were measured in DNA harvested from wild-type and Δ*uvrC::aadA B. burgdorferi* challenged with 2.5 mM DEA/NO for 4 h. Data represent the mean ± SD of three biological samples. ****P < 0.001 compared with untreated controls.

The lack of DNA deamination or AP-site formation in DEA/NO-treated *B. burgdorferi* suggested that DNA may be innately resistant to RNS-induced damage or that DNA repair systems efficiently eliminated RNS-induced DNA lesions. The NER pathway is highly conserved in prokaryotes and repairs a broad range of DNA lesions. The importance of NER in resistance to RNS has been demonstrated by strains harbouring mutations in *uvrA, uvrB* or *uvrC* genes in *Mycobacterium tuberculosis* and *Coxiella burnetii* which are hypersensitive to killing by acidified NO_2_^-^ (Darwin and Nathan, 2005; Park *et al*., 2010). Recently, the NER pathway was implicated in resistance to UV, mitomycin C and H_2_O_2_ in *B. burgdorferi* (Sambir *et al*., 2011). To determine whether the NER pathway contributes to the resistance of *B. burgdorferi* to RNS, ORF *bb0457* (*uvrC*) (476263–478074) and ORF *bb0836* (*uvrB*) (888297–890318) were removed by allelic exchange and replaced with a *flgB_p_::aadA* streptomycin resistance cassette generating the Δ*uvrC::aadA* and Δ*uvrB::aadA* strains respectively (Fig. 4A and B). The contribution of the NER pathway to RNS resistance was determined by challenging wild-type *B. burgdorferi* cells and Δ*uvrC* cells to increasing concentrations of DEA/NO under microaerobic conditions. The Δ*uvrC* strain was 8.5-fold and 24.5-fold more sensitive to killing by 1.25 mM and 2.5 mM DEA/NO than the wild-type *B. burgdorferi* strain (Fig. 4C). The Δ*uvrB* strain was also hypersensitive to killing by 2.5 mM DEA/NO (100-fold) compared with wild-type cells, indicating that a functional NER pathway contributes to the resistance of *B. burgdorferi* to RNS (Fig. 4D). Corresponding to the increased sensitivity of NER-deficient *B. burgdorferi* to killing by RNS, the spontaneous mutation frequency leading to coumermycin A1 resistance increased by 6.5-fold and 8.7-fold in Δ*uvrC* cells over wild-type *B. burgdorferi* cells following treatment with 0.625 mM and 1.25 mM DEA/NO respectively (Fig. 4E). Likewise, *uvrC*-deficient cells displayed a nearly threefold increase in the number of AP sites in their DNA compared with wild-type controls (Fig. 4F). These results indicate that *B. burgdorferi* DNA is damaged by RNS; however, the activity of DNA repair systems including the NER pathway efficiently removes RNS-catalysed DNA lesions in wild-type cells. The efficient removal of DNA lesions from DEA/NO-treated cells suggests that damage to alternative intracellular biomolecules underlies RNS-dependent killing of wild-type *B. burgdorferi*.

### *B. burgdorferi* proteins are susceptible to nitrosative but not nitrative damage

**Fig. 5:**
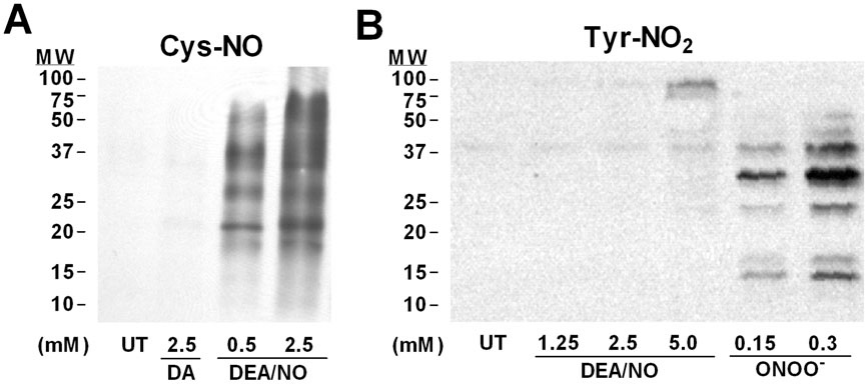
DEA/NO-treated *B. burgdorferi* show *S*-nitrosylation of proteins but lack tyrosine nitration. A. *B. burgdorferi* cells were cultured in the presence or absence of 2.5 mM diethylamine, 0.5 mM DEA/NO or 2.5 mM DEA/NO for 4 h. The biotin-switch technique was used to label S-nitrosylated proteins, which were detected by immunoblot. B. Immunoblot for nitrotyrosine (Tyr-NO_2_) was performed on cell lysates of *B. burgdorferi* cultures exposed to various concentrations of DEA/NO and authentic ONOO^-^ for 4 h.

The lack of damage to cell membranes or DNA suggested that alternative cellular targets were responsible for RNS toxicity in wild-type *B. burgdorferi*. RNS are capable of damaging or modifying cellular proteins in several ways including *S*-nitrosylation of cysteine thiols or nitration of tyrosine residues. *S*-nitrosylation is the reversible addition of a −NO group to free or metal-bound cysteine thiols, which has been shown to modify or inhibit the function of a number of eukaryotic and prokaryotic proteins. The biotin-switch method (BSM) (Jaffrey and Snyder, 2001) was developed to detect S-nitrosylated proteins by (i) blocking unmodified, ‘free’ cysteine thiols via alkylation with either *N*-ethyl maleimide (NEM) or *S*-methyl methanethiosulphonate (MMTS), (ii) the reduction of *S*-nitrosothiols (S-NO) with ascorbate and (iii) the S-biotinylation of the newly reduced cysteine residues using biotin-HPDP. The extent of *S*-nitrosylation is determined by the degree of biotinylation detected by immunoblot. Therefore, the BSM was used to determine the magnitude of *S*-nitrosylation in *B. burgdorferi* cells left untreated to those treated with 2.5 mM diethylamine, 0.5 mM DEA/NO or 2.5 mM DEA/NO (Fig. 5A). While biotinylated proteins were absent in both untreated cells and cells incubated with 2.5 mM diethylamine, a dose-dependent increase in biotinylation was observed in cells exposed to 0.5 mM and 2.5 mM DEA/NO (Fig. 5A). These data indicate that *B. burgdorferi* cells harbour several proteins with cysteine thiols that are susceptible to *S*-nitrosylation by RNS.

The autooxidation of NO can give rise to highly toxic RNS such as NO_2_^•^ and ONOO^-^ that are both potent oxidants and are capable of nitrating tyrosine residues. Nitrotyrosine is a reliable signature for nitrative stress caused by NO_2_^•^ and ONOO^-^. To investigate whether the antimicrobial activity of DEA/NO was linked to nitrative stress, nitrotyrosine formation was assessed in *B. burgdorferi* cells exposed to 1.25–5.0 mM DEA/NO or to 150–300 µM ONOO^-^ for 4 h (Fig. 5B). *B. burgdorferi* cells exposed to DEA/NO did not show significant nitrotyrosine staining. In contrast, cells exposed to ONOO^-^ displayed multiple nitrated proteins (Fig. 5B). Collectively, these data reveal that the susceptibility of wild-type *B. burgdorferi* to killing by DEA/NO is due to nitrosative (*S*-nitrosylation of Cys), rather than nitrative stress (tyrosine nitration).

### NO damages *B. burgdorferi* zinc metalloproteins

**Fig. 6:**
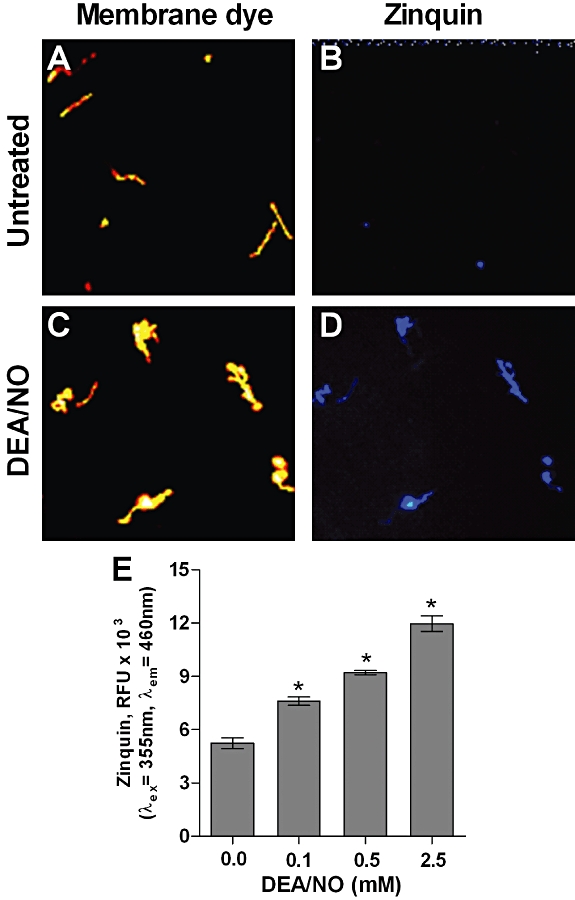
Zinc mobilization in DEA/NO-treated *B. burgdorferi*. *B. burgdorferi* were incubated in the presence or absence of 2.5 mM DEA/NO for 4 h. Untreated cells (A) and 2.5 mM DEA/NO-treated cells (B) were stained with Red Fluorescent Cell Linker Dye. Damage to zinc metalloproteins was determined by monitoring fluorescence emitted by cells incubated for 30 min with Zinquin ethyl ester by fluorescent microscopy (C and D) or by fluorometry (E). Zinquin fluorescence is represented by the mean ± SD of three biological samples. *P < 0.0001 compared with untreated controls.

Zinc is required for *B. burgdorferi* growth and is used as a cofactor for a number of essential enzymes (Posey and Gherardini, 2000). Zinc metalloproteins are well-established targets of RNS in a variety of eukaryotic cells, as well as in the enteric pathogens *E. coli* and *Salmonella enterica* serovar Typhimurium (Kroncke *et al*., 1994; Laval and Wink, 1994; Graziewicz *et al*., 1996; Berendji *et al*., 1997; Aravindakumar *et al*., 1999; Kroncke *et al*., 2001; Schapiro *et al*., 2003). RNS-dependent damage to zinc metalloproteins occurs through *S*-nitrosylation of cysteine thiols that comprise zinc-binding motifs. The zinc-dependent fluorophore Zinquin has been used as an indicator of damage to zinc metalloproteins by monitoring the NO-dependent mobilization of zinc in *Salmonella typhimurium* and *E. coli* (Binet *et al*., 2002; Schapiro *et al*., 2003). To determine whether zinc metalloproteins are similarly targeted by RNS in *B. burgdorferi*, zinc mobilization was compared in untreated cells and cells treated with 2.5 mM DEA/NO. DEA/NO-treated *B. burgdorferi* displayed robust Zinquin-dependent fluorescence compared with untreated controls (Fig. 6B and D). Moreover, the magnitude of intracellular Zinquin fluorescence was enhanced by increasing concentrations of DEA/NO (Fig. 6E), indicating that zinc metalloproteins are major targets of RNS in *B. burgdorferi*.

### Identification of *B. burgdorferi* proteins targeted by RNS

**Fig. 7:**
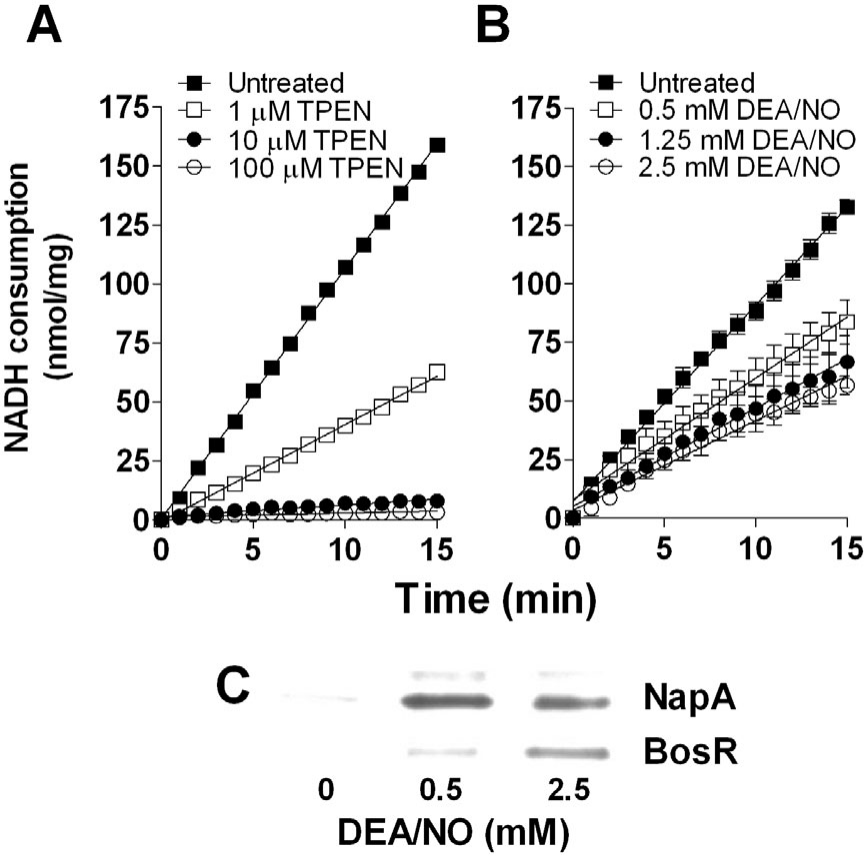
*BB0445*, BosR and NapA are targets of RNS. A. Aldolase activity was measured from *B. burgdorferi* crude cell lysates exposed to various concentrations of the zinc-chelator TPEN for 10 min. B. Aldolase activity of crude cell lysates generated from *B. burgdorferi* cells treated with 0, 0.5, 1.25 or 2.5 mM DEA/NO for 30 min. Aldolase activity is represented by the mean consumption of NADH (nmol) per mg protein ± SD of four biological samples. C. *S*-nitrosylation of BosR and NapA in *B. burgdorferi* cells exposed to 0, 0.5 or 2.5 mM DEA/NO for 4 h is shown for separate Western blots using either α-BosR or α-NapA polyclonal antibodies against the affinity-purified biotinylated proteins generated using the biotin-switch method shown in Fig. 5A.

Assays for *S*-nitrosylation and zinc mobilization showed that several *B. burgdorferi* proteins are subject to nitrosative modification in DEA/NO-treated cells. *B. burgdorferi* encodes several putative zinc metalloproteins; however, only peptide deformylase and the transcriptional regulator BosR have been formally characterized as zinc-binding proteins (Boylan *et al*., 2003; Nguyen *et al*., 2007). Open reading frame *bb0445* is annotated as a putative class II, zinc-dependent, fructose-1,6-bisphosphate aldolase that functions in the glycolytic cycle of *B. burgdorferi* (Fraser *et al*., 1997). Crude cell lysates of *B. burgdorferi* showed fructose-1,6-bisphosphate aldolase activity (Fig. 7A and Table 1). Consistent with the zinc-dependence of class II, fructose-1,6-bisphosphate aldolases, exposure of cell lysates to 10–100 µM of the zinc-chelator N,N,N′,N′-tetrakis-(2-pyridylmethyl)ethylenediamine (TPEN) for 10 min reduced aldolase activity by more than 95% (Table 1). In order to determine whether BB0445 (Fba) was a target of RNS, aldolase activity was measured from *B. burgdorferi* cells treated with increasing concentrations of DEA/NO for 30 min. Cells treated with 0.5–2.5 mM DEA/NO showed a dose-dependent reduction in aldolase activity compared with untreated controls (Fig. 7B and Table 1). RNS-dependent inhibition of aldolase activity reached 57.3% in cells treated with 2.5 mM DEA/NO (Table 1). The DEA/NO-dependent inhibition of aldolase activity was consistent with RNS-induced damage to zinc metalloproteins seen in Fig. 6, as well as the requirement for zinc for aldolase activity (Fig. 7A). Collectively, these data identify BB0445 as a zinc metalloprotein targeted by RNS in *B. burgdorferi*, whose inhibition may have significant consequences for cellular energy and metabolism.

**Table 1:** *B. burgdorferi* fructose-1,6-bisphosphate aldolase activity

	Aldolase activity (U mg^−1^)		
		
	Average	SD	% Inhibition
TPEN			
Untreated	0.0106	0.0001	–
1 µM	0.0042*	0.0001	60.6
10 µM	0.0005*	0.0001	95.9
100 µM	0.0002*	0.0001	97.9
DEA/NO			
Untreated	0.0089	0.0002	–
0.5 mM	0.0056**	0.0006	37.0
1.25 mM	0.0044**	0.0007	49.7
2.50 mM	0.0038**	0.0002	57.3

One unit (U) of activity = consumption of 1 µmol NADH min^−1^.

% Inhibition = aldolase activity of treated samples/untreated samples.

* *P* < 0.05 compared with untreated controls.

** *P* < 0.05 compared with untreated controls.

BosR is a zinc-dependent transcriptional activator that harbours two pairs of cysteines. One pair, Cys-114 and Cys-117, are hypothesized to bind zinc, while Cys-153 and Cys-156 may act as a redox switch (Boylan *et al*., 2003). BosR regulates the expression of NapA, which encodes a Cys-rich, C-terminal tail that likely serves as a zinc-binding domain. The fact that both BosR and NapA harbour potentially redox-active cysteine residues that may be involved in zinc binding, combined with their important role in the antioxidant defences of *B. burgdorferi*, suggested they may be prime targets for *S*-nitrosylation. To determine whether BosR and NapA were targeted by RNS *B. burgdorferi* cells were incubated with 0, 0.5 and 2.5 mM DEA/NO for 4 h and S-nitrosylated proteins were derivatized using the BSM as in Fig. 5A. Biotinylated proteins were affinity-purified using streptavidin agarose and separated by SDS-PAGE. Western blots using α-NapA and α-BosR antibodies revealed that NapA and BosR were among the affinity-purified biotinylated proteins, indicating that both proteins were S-nitrosylated in *B. burgdorferi* cells exposed to DEA/NO (Fig. 7C). Collectively, these data identify Fba, BosR and NapA as specific proteins targeted by RNS in *B. burgdorferi*.

## Discussion

RNS contribute to innate host defences against diverse microbial pathogens; however, only a few bacterial species have been shown to be directly killed by RNS produced *in vitro*. For example, *M. tuberculosis* is exquisitely sensitive to killing by RNS generated by acidified NO_2_ and other NO donors (Long *et al*., 1999; Darwin *et al*., 2003), while the zoonotic pathogen *Burkholderia mallei* is killed by treatment with the NO donor spermine NONOate (Jones-Carson *et al*., 2008). Preliminary studies on the overall bactericidal effect of RNS on *B. burgdorferi* confirmed previous reports that the cells were sensitive to NO generated *in vitro* (Ma *et al*., 1994; Lusitani *et al*., 2002). These results raised very interesting questions given the innate resistance of *B. burgdorferi* to killing by ROS. Previous work from our laboratory showed that *B. burgdorferi* cell membranes were damaged ROS, while crucial intracellular targets (i.e. DNA) were unaffected by treatment with concentrations of H_2_O_2_ or t-butyl peroxide reaching 10 mM (Boylan *et al*., 2008). This innate resistance likely stems from the lack of intracellular iron and iron-dependent proteins required for the production of harmful Fenton-derived OH^•^, as well as the activity of antioxidant defences such as SodA (Posey and Gherardini, 2000; Esteve-Gassent *et al*., 2009). The fact that ROS and RNS share many of the same intracellular biomolecular targets (e.g. DNA) suggested that the observed differences in sensitivity between ROS and RNS in *B. burgdorferi* could be attributed to differences in the chemical modifications of biomolecular targets (i.e. oxidation versus deamination of DNA bases) or differences in the repair mechanisms of specific lesions.

While the antimicrobial activity of RNS is well established, the mechanisms by which RNS kill bacteria are not well understood. DNA damage is a probable mechanism, since RNS generate DNA lesions including deamination, formation of abasic sites and strand breaks, and other chemical modifications of DNA *in vitro* (Tamir *et al*., 1996; Burney *et al*., 1999). Our data indicate that DNA is a target of RNS in *B. burgdorferi*, as both *uvrB*- and *uvrC*-deficient cells showed an increased susceptibility to killing by RNS compared with wild-type controls. However, RNS-induced DNA lesions are efficiently removed by DNA repair machinery including the NER pathway, as wild-type *B. burgdorferi* exposed to lethal concentrations of DEA/NO showed no indication of deamination or increased numbers of AP sites. This observation was consistent with the absence of DNA damage in ROS-challenged *B. burgdorferi* (Boylan *et al*., 2008). The absence of ROS-induced DNA damage was attributed to a lack of intracellular targets (i.e. Fe–S clusters) or the limited potential for production of Fenton-derived OH^•^ radicals; however, a recent study demonstrated that a functional *uvrA* (*bb0837*) allele is required to protect *B. burgdorferi* DNA against damage caused by ROS, mitomycin C and UV (Sambir *et al*., 2011). These findings, coupled with our observations with both *uvrB-* and *uvrC*-deficient strains, reveal an unequivocal role for the NER pathway in the overall resistance of *B. burgdorferi* DNA to oxidative and nitrosative damage. Other DNA repair pathways encoded by the *B. burgdorferi* genome (Fraser *et al*., 1997) including the BER pathway [uracil DNA glycosylase (*ung*, *bb0053*); 3-methyladenine glycosylase (*mag*, *bb0422*); endonuclease III (*nth, bb0745*); and exodeoxyribonuclease III (*exoA*, *bb0534*)], the mismatch repair pathway [*mutS* (*bb0797*), *mutS-II* (*bb0098*) and *mutL* (*bb0211*)], as well as DNA recombinase A (*recA, bb0131*) may also play important roles in defending against the genotoxic effects of ROS and RNS.

Akin to the lack of DNA damage, exposure of *B. burgdorferi* cells to DEA/NO failed to stimulate lipid peroxidation, as indicated by the lack ‘membrane blebs’ characteristic of ROS-induced damage or increases in the lipid peroxidation end-product MDA. This finding was surprising given the susceptibility of polyunsaturated fatty acids to peroxidation by RNS such as NO_2_^•^ and ONOO^-^ in both prokaryotic and eukaryotic systems (Freeman *et al*., 2008). The lack of DEA/NO-catalysed lipid peroxidation in *B. burgdorferi* may be explained by the cytoprotective effects of NO, which pairs with lipid peroxyl adducts to terminate lipid peroxidation (Freeman *et al*., 2008). Our results do not preclude the ability of RNS to catalyse nitrosative or nitrative modifications of polyunsaturated fatty acids in *B. burgdorferi* cell membranes. However, exposure of *B. burgdorferi* to lethal concentrations of DEA/NO did not result in a loss of membrane integrity, suggesting that the antimicrobial effects of RNS are independent of damage to lipid membranes.

While membrane lipids are unaffected and DNA damage is efficiently repaired in wild-type *B. burgdorferi* exposed to lethal doses of RNS, free and zinc-bound cysteine thiols are highly susceptible to nitrosative damage. Using the biotin-switch technique (Jaffrey and Snyder, 2001), we showed that exposure of *B. burgdorferi* to DEA/NO under microaerobic conditions produced a nitrosative environment that resulted in the *S*-nitrosylation of several proteins, including BosR and NapA. The *S*-nitrosylation of BosR by RNS is intriguing given its role in co-ordinating the expression of antioxidant defence genes such as *cdr*, *napA* and *sodA*, as well as the virulence genes *rpoS*, *ospC* and *dbpA* (Boylan *et al*., 2003; 2006; Hyde *et al*., 2006; 2009; 2010; Ouyang *et al*., 2009). BosR harbours four Cys residues that comprise two distinct CXXC motifs. Cys-114 and Cys-117 hypothetically serve as a zinc-binding domain, while Cys-153 and Cys-156 may comprise a redox switch. However, the importance of these cysteine residues to BosR function has yet to be determined. *S*-nitrosylation or oxidation of Cys-153 or Cys-156 by RNS may induce conformational changes to BosR akin to those of OxyR shifting its activity to a transcriptionally active form (Kim *et al*., 2002; Boylan *et al*., 2003). Based on the sensitivity of zinc metalloproteins to damage by RNS (Fig. 6), it is tempting to speculate that at high concentrations of RNS may disrupt the hypothetical Cys-114/117 zinc-binding motif of BosR resulting in its release from cognate promoters. Effects of RNS on BosR-dependent gene transcription, the characterization of the roles Cys-114, -117, -153 and -156 play in BosR function, and the identification of the specific cysteine residue(s) subject to *S*-nitrosylation will help shed light on the roles of both BosR and RNS in *B. burgdorferi* pathogenesis.

NapA is a Dps-like, membrane-associated protein whose expression is regulated by BosR. NapA harbours a unique poly-Cys tail at its C-terminus that may serve a catalytic function and is predicted to serve as a key defence against ROS-mediated lipid peroxidation in the *B. burgdorferi* cell envelope. The poly-Cys tail may serve as a zinc-binding domain, although it is not yet known whether zinc is required for NapA enzymatic activity. Our data reveal that NapA is among the proteins susceptible to *S*-nitrosylation and suggest that its regulation by BosR may be a component of the antioxidative/antinitrosative defences of *B. burgdorferi*. Of particular interest is whether the C-terminal, poly-Cys tail of NapA serves as a sink for RNS at the *B. burgdorferi* cell membrane, which may explain why treatment with RNS failed to elicit membrane damage akin to treatment with ROS.

In addition to BosR and NapA, this study identified fructose-1,6-bisphosphate aldolase (BB0445) as a zinc metalloenzyme that is targeted by RNS. However, the mechanism underlying nitrosative inhibition of this enzyme has yet to be determined. BB0445 encodes four cysteines that are potential targets for *S*-nitrosylation, of which Cys-36 and Cys-111 have been characterized in the homologous fructose-1,6-bisphosphate aldolase of *E. coli*. While Cys-36 has been shown to be surface-exposed and reactive in *E. coli* (Packman and Berry, 1995), it does not appear to be required for aldolase enzymatic activity making it an unlikely candidate for the nitrosative inhibition of BB0445. In contrast, the *S*-nitrosylation of Cys-111 in BB0445 may have a detrimental effect on aldolase activity. Site-directed mutagenesis of Cys-112 (Cys-111 in *B. burgdorferi*) of fructose-1,6-bisphosphate aldolase in *E. coli* reduced the number zinc ions per dimer by > 50% and resulted in a 70% reduction in aldolase activity (Berry and Marshall, 1993), which is similar to the degree of nitrosative inhibition of BB0445 in DEA/NO-treated *B. burgdorferi* cells. Further investigation will be required to elucidate the direct effects of RNS on BB0445 function and zinc binding.

Because *B. burgdorferi* lacks enzymes involved in oxidative phosphorylation or the TCA cycle and relies on glycolysis for ATP production (Fraser *et al*., 1997), the inhibition of BB0445 by RNS may have significant effects on cellular metabolism. Inhibition of fructose-1,6-bisphosphate aldolase may result in a significant reduction in the cytoplasmic ATP reservoir required for fuelling a multitude of cellular reactions such as the V_0_V_1_ ATPase that maintains proton motive force. These findings also raise the intriguing possibility that this enzyme may be a ubiquitous target of RNS in bacterial pathogens such as *S. typhimurium* that encounter RNS throughout their pathogenic cycle. Interestingly, *S. typhimurium* encodes two fructose-1,6-bisphosphate aldolases, a class II, zinc-dependent homologue to that of *B. burgdorferi*, as well as a class I, zinc-independent aldolase that catalyses the aldol reaction using a reactive lysine residue. The presence of the class I, fructose-bisphosphate aldolase may provide a mechanism by which *S. typhimurium* is able to sustain its glycolytic pathway following challenge with RNS that may result in the inactivation of the class II, zinc-dependent aldolase. This may represent a similar strategy to that employed by *E. coli* which encodes two aconitases *acnA* and *acnB*, which exhibit differential susceptibilities to inactivation by ROS (Varghese *et al*., 2003). Consequently, *E. coli* is able to maintain a functional TCA cycle with the O_2_^•−^-resistant AcnA, despite the O_2_^•−^-dependent inactivation of the more highly expressed and efficient AcnB.

**Fig. 8:**
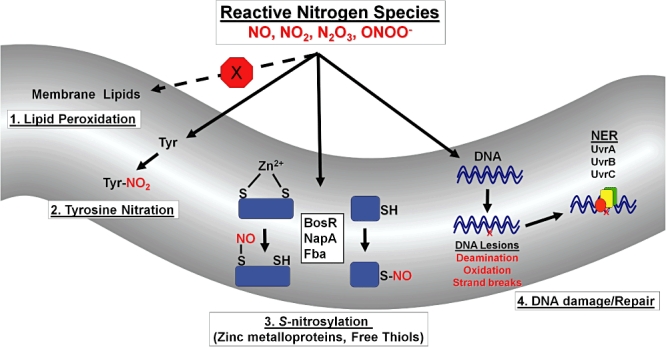
Proposed molecular targets of RNS in *B. burgdorferi*. Throughout its infectious cycle *B. burgdorferi* encounters RNS capable of modifying or damaging diverse biomolecules. 1. Unlike ROS, RNS do not trigger lipid peroxidation of polyunsaturated fatty acids in the *B. burgdorferi* cell membrane. 2. Tyrosine residues of *B. burgdorferi* proteins are susceptible to nitration by ONOO^-^, but not by the RNS produced by DEA/NO. 3. Free and zinc-bound cysteine thiols are primary targets of RNS in *B. burgdorferi*. This study has identified BosR, NapA and the *BB0445*-encoded fructose-1,6-bisphosphate aldolase (Fba) as specific targets of RNS. 4. *B. burgdorferi* DNA damaged by RNS is efficiently repaired by DNA repair systems including the nucleotide excision repair (NER) pathway (UvrA, UvrB and UvrC).

This study sought to identify the mechanism(s) underlying RNS toxicity in *B. burgdorferi*. In contrast to ROS, the antimicrobial effects of RNS against *B. burgdorferi* appear to be independent of damage to cell membranes by lipid peroxidation (Boylan *et al*., 2008). Rather, our results have led us to a model (Fig. 8) in which *B. burgdorferi* proteins harbouring free and zinc-bound cysteine thiols are the major targets of RNS. We have identified three proteins specifically targeted by RNS: the transcriptional regulator BosR, the membrane protein NapA and the glycolytic enzyme, Fba (BB0445). This study has also revealed that *B. burgdorferi* harbours proteins that are susceptible to tyrosine nitration when RNS such as NO_2_^•^ or ONOO^-^ are present; however, this modification is not apparent in cells exposed to lethal concentrations of DEA/NO (Fig. 5B). Our data also indicate that DNA is a target of RNS in *B. burgdorferi*; however, RNS-induced DNA lesions (i.e. deamination, oxidation, strand breaks) do not appear to facilitate killing of wild-type *B. burgdorferi* due to their efficient removal by NER proteins (UvrA, UvrB and UvrC) and other DNA repair pathways.

In summary, we have distinguished between the cellular targets of RNS (Cys thiols, DNA) and ROS (cell membranes) in *B. burgdorferi*. The fact that ROS and RNS have distinct cellular targets in *B. burgdorferi* suggests that their contribution to host defence may be greatest when both are present. At this point, roles for either ROS or RNS in mammalian host defences against *B. burgdorferi* are limited to their roles in the borreliacidal activity of macrophages (Ma *et al*., 1994; Modolell *et al*., 1994; Lusitani *et al*., 2002). However, ongoing studies in our laboratory suggest that ROS and RNS present in *I. scapularis* salivary glands may represent significant obstacles to *B. burgdorferi* during transmission to its mammalian hosts.

## Experimental procedures

### Strains, growth conditions and reagents

Low-passage *B. burgdorferi* strain B31 A3 was grown in BSK-II medium (Barbour *et al*., 1984) under microaerobic (3% O_2_, 5% CO_2_) or anaerobic conditions (5% CO_2_, 4% H_2_, balance N_2_) and cell density was determined by dark-field microscopy. *E. coli* strain TA4315 (Δ*ahpCF*) (Storz *et al*., 1989) was grown in Luria–Bertani (LB) broth at 37°C with shaking unless otherwise noted.

Deletions of NER genes *uvrC* (*bb0457*) and *uvrB* (*bb0836*) were achieved by homologous recombination using suicide vectors pPCR-Script Cam::Δ*uvrC::aadA* (pCm::Δ*uvrC*) and pPCR-Script Cam::Δ*uvrB::aadA* (pCm::Δ*uvrB*). All PCR products used to generate pCm::Δ*uvrC* and pCm::Δ*uvrB* were generated using La *Taq* polymerase (Takara Bio, Madison, WI) and primers with engineered restriction sites (Table 2). Briefly, 1 kb regions upstream and downstream of either *uvrC* or *uvrB* were amplified by PCR from wild-type *B. burgdorferi* B31 A3 DNA. The *aadA* streptomycin resistance cassette under the control of a *flgB* promoter (*flgB_P_-aadA*) was amplified by PCR from vector pKFSS1 using primers aadA-ClaI-F and aadA-SacII-R (Table 2). The PCR product encoding the 1 kb region upstream of *uvrC* or *uvrB* was cloned into pPCR-Script Cam SK(+) (Agilent Technologies, Cedar Creek, TX) using engineered restriction sites for KpnI and ClaI, followed by the PCR products encoding the *aadA* cassette using ClaI and SacII, and the 1 kb region downstream of *uvrC* or *uvrB* using SacII and SacI to generate pCm::Δ*uvrC*. Twenty micrograms of pCm::Δ*uvrC* or pCm::Δ*uvrB* was electroporated into *B. burgdorferi* B31 A3 with transformants selected for by plating on BSK II medium supplemented with 40 µg ml^−1^ streptomycin. PCR was used to verify Δ*uvrC::aadA* and Δ*uvrB::aadA* clones using the primers listed in Table 2.

**Table 2:** Strains, plasmids and primers

Strains		Reference
*Borrelia burgdorferi* B31 A3		Elias *et al*. (2002)
*Borrelia burgdorferi* B31 A3 Δ*uvrC::aadA*		This study
*Borrelia burgdorferi* B31 A3 Δ*uvrB::aadA*		This study
*Escherichia coli* TA4315 (*ahpCF^-^*)		Storz *et al*. (1989)

Plasmids		Reference
pKFSS1		Frank *et al*. (2003)
pPCR-Script Cam SK(+)		Agilent
pCm::Δ*uvrC::aadA*		This study
pCm::Δ*uvrB::aadA*		This study

Primer name	Sequence (5′–3′)	Reference
aadA-ClaI-F	ACTGTAATCGATTACCCGAGCTTCAAGGAA	This study
aadA-SacII-R	ACTGTACCGCCGTATTTGCCGACTACCTTG	
uvrC-KpnI-F	ACTGTAGGTACCAGCACTACTAACCTCTTC	
uvrC-ClaI-R	ACTGTAATCGATTCTTTCATAATGTTAGGCAG	This study
uvrC-SacII-F	ACTGTACCGCGGTTGCAATAAAAGAGAACTCC	This study
uvrC-SacI-R	ACTGTAGAGCTCAAGATATGACTCAGTCTTTG	This study
uvrB-KpnI-F	ACTGTAGGTACCTGTTGCAAGGGCAATGTCAC	
uvrB-ClaI-R	ACTGTAATCGATTCTCTTTTATTGCCTTAGGC	This study
uvrB-SacII-F	ACTGTACCGCGGTGTCAGAGGAGCAAAAGAAC	This study
uvrB-SacI-R	ACTGTAGAGCTCAGCCACAGACCAAGATGAAC	This study

The underlined sequence denotes the restriction endonuclease site of the primer.

### *In vitro* sensitivity to RNS

Fifty millilitres of BSK-II medium was inoculated with *B. burgdorferi* strain B31 A3 and the cultures were grown under microaerobic or anaerobic conditions to a cell density of 5 × 10^7^ cells ml^−1^. Next, the cultures were split and challenged with varying concentrations of the NO donor DEA/NO (Cayman Chemical, Ann Arbor, MI). Following treatment, the cells were diluted, plated on BSK-II media and incubated at 34°C for 7–14 days. Per cent survival was calculated by dividing the number of colonies on the treated plates by the number of colonies on untreated plates.

### Determination of lipid damage

Electron microscopy was carried out on late-log-phase cultures of anaerobically grown *B. burgdorferi* exposed to 0 mM, 2.5 mM and 5.0 mM diethylamine NONOate at 34°C for 4 h as previously described (Boylan *et al*., 2008). Cells were examined using a Hitachi H7500 electron microscope (Hitachi High Technologies America, Pleasanton, CA).

The amount of the peroxidation end-product MDA was measured as previously described (Boylan *et al*., 2008) from lipids extracted from microaerobic cultures of *B. burgdorferi* strain B31A3 treated with 5.0 mM DEA/NO or 1 mg ml^−1^ (158 000 U ml^−1^) lipoxidase (Sigma, St. Louis, MO) at 34°C for 4 h using the Microplate Assay for Lipid Peroxidation per manufacturer's instructions (Oxford Biomedical Research, Oxford, MI).

### Measurement of DNA damage

*Borrelia burgdorferi* B31 A3 were treated with 0 mM, 2.5 mM and 5.0 mM DEA/NO under microaerobic conditions at 34°C for 4 h. Total DNA was isolated using the DNeasy Blood and Tissue Kit (Qiagen, Valencia, CA). The number of AP sites in the harvested DNA samples was quantified using the DNA Damage Quantitation Colorometric Assay kit (Oxford Biomedical Research) following the manufacturer's protocol. Briefly, 500 ng of DNA was incubated with an aldehyde reactive probe (ARP) at 37°C for 1 h. The DNA–ARP mixture was combined with glycogen solution, ethanol precipitated, washed three times with 70% ethanol and resuspended in Tris-EDTA to 0.5 µg ml^−1^. The DNA–ARP samples were added to a 96-well plate with DNA binding buffer and allowed to bind for 12 h at room temperature. Wells were washed four times with TPBS (137 mM NaCl, 2.7 mM KCl, 10 mM Na_3_HPO_4_, 2.5 mM KH_2_PO_4_, 0.5% Tween 20, pH 7.4). Following washing, 100 µl of the HRP-streptavidin conjugate diluted in TPBS was added to each well and incubated at room temperature for 1 h. Wells were washed five times with TPBS, 100 µl of substrate added to each well and incubated for 1 h at 37°C. After incubation, 100 µl of 1 M H_2_SO_4_ was added to each well to quench the reaction. Samples were read at 450 nm and the quantity of aldehyde reactive probe (ARP) DNA base lesions was determined using a standard curve. *E. coli* strain TA4315 (Δ*ahpCF*) (Storz *et al*., 1989) was used as a positive control for DNA damage. DNA was harvested and the number of AP sites measured from strain TA4315 grown to mid-log phase (OD_600_ = 0.4) in minimal M9 medium + 0.2% glucose and challenged with 5.0 mM H_2_O_2_ or 2.5 mM DEA/NO for 1 h at 37°C (Boylan *et al*., 2008). DNA damage was determined as described above.

DNA from microaerobic *B. burgdorferi* cultures exposed to DEA/NO was harvested as described above and concentrations of deoxyinosine were determined by HPLC. Briefly, 1 µg of heat-denatured DNA was digested with P1 nuclease (United States Biological, Swampscott, MA) at 37°C for 2 h, followed by incubation with 10 U of Antarctic phosphatase (New England Biolabs, Ipswich, MA) at 37°C for 20 h. Digested DNA samples were injected onto a Supelcosil LC-18-S 4.6 × 150 mm, 5 µm analytical column (Sigma) pre-equilibrated with 50 mM potassium phosphate, pH 4.5 containing 8% methanol. The column was eluted for 20 min at a rate of 1 ml min^−1^ and monitored for absorbance at 254 nm. The composition of each peak was identified by comparison to authentic deoxynucleoside standards (Sigma). The quantity of deoxyinosine was determined by comparison to a standard curve. The data were derived from three biological samples measured in triplicate.

The spontaneous mutation rate to coumermycin A1 was determined for wild-type *B. burgdorferi* B31 A3 and the *uvrC*-deficient strain (B31 A3 Δ*uvrC::aadA*) treated with 0 or 0.625 mM DEA/NO at 34°C for 4 h. Following treatment with DEA/NO cells were plated on BSK-II plates containing 0 or 250 ng ml^−1^ coumermycin A1 and incubated for 14 days. The resistance frequency was calculated by dividing the number of Cou^r^ cfu by the number cfu on BSK-II plates without coumermycin A1. The data were derived from three biological replicates for each treatment.

### Detection of S-nitrosylated and nitrated proteins

The biotin-switch technique was used to detect S-nitrosylated proteins (Jaffrey and Snyder, 2001) with slight modifications. Ten millilitres of cultures of *B. burgdorferi* B31 A3 were treated with various concentrations of DEA/NO or diethylamine for 4 h under microaerobic conditions. Cells were pelleted by centrifugation (4000 r.p.m. for 10 min at 4°C), washed 2 × 1 ml of HN (20 mM NaCl, 50 mM HEPES, pH 7.6) buffer and lysed in 1 ml of HENS blocking buffer (1 mM DPTA, 0.1 mM neocuproine, 2.5% SDS, 0.5% Triton X-100, 0.1 mM NEM, 250 mM HEPES, pH 7.7). Cell lysates in HENS blocking buffer were incubated in a dark room at 50°C for 30 min with frequent vortexing to block unmodified, free cysteine thiols by alkylation with NEM. Following blocking, proteins were precipitated with 4 ml of ice-cold acetone and placed at −20°C for 1 h. Proteins were pelleted by centrifugation (6000 r.p.m. for 10 min at 4°C) and washed in 3 × 5 ml of acetone. S-nitrosylated proteins were reduced using sodium ascorbate and *S*-biotinylated with biotin-HPDP by dissolving the precipitated proteins in 1 ml of HENS biotinylation buffer (0.4 mM biotin-HPDP, 1% SDS, 1 µM Cu^II^ sulphate, 5 mM sodium ascorbate, 250 mM HEPES, pH 7.7) and incubated in the dark at room temperature for 1 h. Following biotinylation, proteins were precipitated with 4 ml of ice-cold acetone at −20°C for 1 h. Proteins were pelleted by centrifugation and washed in 3 × 5 ml of acetone. Pelleted proteins were dried at room temperature and dissolved in 1 ml of PBS. Protein concentrations were determined using the BCA protein assay kit (Pierce) according to the manufacturer's instructions. Protein concentrations were normalized to 500 µg ml^−1^ and samples separated on non-reducing, SDS/10% polyacrylamide gels. Proteins were transferred to nitrocellulose membranes and blocked overnight at 4°C with 2% BSA in TBST (0.1% Tween-20, 150 mM NaCl, 100 mM Tris, pH 7.5) buffer. Blocked membranes were probed with a 1:3000 dilution of streptavidin-HRP (Pierce). The membranes were washed five times in TBST and immunoreactive proteins were visualized as described in the technical manual for the Enhanced Chemiluminescence (ECL) Kit (GE Healthcare, Piscataway, NJ).

The biotin-switch protein samples generated above were used to affinity purify biotinylated proteins by adding 200 µl of protein samples (500 µg ml^−1^) to 100 µl of streptavidin agarose resin (Thermo) and mixing at room temperature for 1 h. Streptavidin agarose was pelleted by centrifugation (5000 r.p.m. for 1 min) and washed with 3 × 1 ml of PBS. The streptavidin agarose mixture was dissolved in 200 µl of Laemmli sample buffer + 2-mercaptoethanol and boiled for 5 min. Proteins were separated on SDS/15% polyacrylamide gels and transferred to nitrocellulose membranes. Membranes were blocked overnight at 4°C with 5% non-fat dry milk in TBST buffer. Membranes were probed with 1:500 dilutions of either the α-BosR or α-NapA polyclonal antibodies (Boylan *et al*., 2003) at room temperature for 1 h and washed five times with TBST. The membranes were incubated with a 1:2000 dilution of an HRP-conjugated goat anti-rabbit IgG secondary antibody in TBST at room temperature for 1 h. The membranes were washed five times with TBST and immunoreactive proteins visualized using the ECL Kit as described above.

Nitrotyrosine formation was determined for microaerobic cultures of *B. burgdorferi* exposed to specified amounts of DEA/NO or authentic ONOO^-^ (Mohr *et al*., 1994) for 4 h at 34°C. Cells were harvested by centrifugation (4000 r.p.m. for 5 min), washed twice in HN buffer, and resuspended in 1 ml of HN buffer to a cell density of 5 × 10^7^ cells ml^−1^. Cells were lysed in 2× Laemmli sample buffer + 2-mercaptoethanol and proteins were separated on 10% SDS/polyacrylamide gels. Proteins were transferred to nitrocellulose and probed with a 1:5000 dilution of the anti-nitrotyrosine polyclonal antibody (Upstate, Lake Placid, NY). Nitrocellulose membranes were incubated in TBST with a 1:10 000 dilution of an HRP-conjugated goat anti-rabbit IgG secondary antibody for 1 h, washed five times with TBST and immunoreactive proteins were visualized as described above. Relative sample loading was assessed by staining duplicate 10% SDS/ polyacrylamide gels with Coomassie Brilliant Blue stain.

### Measurement of damage to zinc metalloproteins

Late-log-phase *B. burgdorferi* cultures were pelletted by centrifugation (1000 *g,* 10 min, 4°C), washed 2× in HN buffer and resuspended in HN buffer + 25 µM Zinquin ethyl ester (Sigma) and incubated at 34°C for 30 min under microaerobic conditions. Cells pelleted by centrifugation and washed 2× in HN buffer to remove extracellular Zinquin. Following resuspension in HN buffer cells were exposed to DEA/NO for 30 min. Zinquin fluorescence was monitored (Ex_λ_ = 355 nm, Em_λ_ = 460 nm) using a Victor3V 1420 multilabel reader (PerkinElmer, Waltham, MA). Alternatively, cells treated with 0 or 2.5 mM DEA/NO for 30 min were pelleted, washed and resuspended in HN buffer + 25 µM Zinquin. Following incubation at 34°C for 30 min cells were labelled with Red Fluorescent Cell Linker Dye (Sigma) and analysed by fluorescent microscopy.

Measurement of fructose-1,6-bisphosphate aldolase (Fba) activity was carried out on microaerobic *B. burgdorferi* cells exposed to specified amounts of DEA/NO for 30 min at 34°C as previously described (Berry and Marshall, 1993) with minor changes. Briefly, cells were pelleted by centrifugation, washed three times with Tris buffer (100 mM, pH 7.2) and lysed by sonication. Protein concentrations were quantified using the BCA protein assay (Pierce, Rockford, IL) and normalized to 100 µg ml^−1^. One millilitre of aldolase reactions consisted of 700 µl of Tris buffer pH 7.2 (100 mM), 100 µl NADH (4 mM), 100 µl of fructose-1,6-bisphosphate (58 mM), 2.5 U of glycerophosphate dehydrogenase type I from rabbit muscle, 2.5 U of triosphosphate isomerase from rabbit and 100 µl of cell lysate (10 µg rxn^−1^). All reagents were from Sigma. Fba activity was determined by monitoring the consumption of NADH at Abs_λ_ = 340 nm for 15 min using a Victor3V 1420 multilabel reader (PerkinElmer). Data were collected from four biological samples measured in duplicate.
